# Enhancing Radiation Detection by Drones through Numerical Fluid Dynamics Simulations

**DOI:** 10.3390/s20061770

**Published:** 2020-03-23

**Authors:** Fabio Marturano, Jean-François Ciparisse, Andrea Chierici, Francesco d’Errico, Daniele Di Giovanni, Francesca Fumian, Riccardo Rossi, Luca Martellucci, Pasquale Gaudio, Andrea Malizia

**Affiliations:** 1 Department of Industrial Engineering, University of Rome Tor Vergata, 00133 Rome, Italy; etribusvis@gmail.com (F.M.); ciparisse@uniroma2.it (J.-F.C.); chieandre@yahoo.it (A.C.); daniele.di.giovanni@uniroma2.it (D.D.G.); francescafumian@gmail.com (F.F.); r.rossi@ing.uniroma2.it (R.R.); lc.martellucci@gmail.com (L.M.); gaudio@ing.uniroma2.it (P.G.); 2 Department of Civil and Industrial Engineering, University of Pisa, 56122 Pisa, Italy; francesco.derrico@ing.unipi.it; 3 Department of Biomedicine and Prevention, University of Rome Tor Vergata, 00133 Rome, Italy

**Keywords:** detection, drone, radiation, simulation, measure, instrumentation

## Abstract

This study addresses the optimization of the location of a radioactive-particle sensor on a drone. Based on the analysis of the physical process and of the boundary conditions introduced in the model, computational fluid dynamics simulations were performed to analyze how the turbulence caused by drone propellers may influence the response of the sensors. Our initial focus was the detection of a small amount of radioactivity, such as that associated with a release of medical waste. Drones equipped with selective low-cost sensors could be quickly sent to dangerous areas that first responders might not have access to and be able to assess the level of danger in a few seconds, providing details about the source terms to Radiological-Nuclear (RN) advisors and decision-makers. Our ultimate application is the simulation of complex scenarios where fluid-dynamic instabilities are combined with elevated levels of radioactivity, as was the case during the Chernobyl and Fukushima nuclear power plant accidents. In similar circumstances, accurate mapping of the radioactive plume would provide invaluable input-data for the mathematical models that can predict the dispersion of radioactivity in time and space. This information could be used as input for predictive models and decision support systems (DSS) to get a full situational awareness. In particular, these models may be used either to guide the safe intervention of first responders or the later need to evacuate affected regions.

## 1. Introduction

The use of drones has increased dramatically over the past two decades, introducing a real paradigm shift in sectors as diverse as the military, medical, commercial, and entertainment. Easily controlled via a base-station or even a smartphone app, they can reach the most remote, hostile or otherwise inaccessible areas, requiring minimum amounts of time/effort and power. An emerging application is monitoring complex radiological/nuclear scenarios such as the 2011 Fukushima Daiichi nuclear plant accident, with a combined large release of heat and nuclear radiation. Accurate mapping of the radioactive plume during the accident would have provided invaluable input-data for the mathematical models that can predict the dispersion of radioactivity in time and space. Since drones are inevitably equipped with point-wise radiation sensors, acquiring high-resolution maps requires close proximity to the source term. On the other hand, launching drones above a heat source causing severe turbulence requires the ability to predict and control their behavior, lest theybelost immediately. The situation is analogous, whenever the accidental or deliberate release of Chemical, Biological, and Radiological (CBR) substances is involved [1,2,3,4]. The Chemical-Biological-Radiological-Nuclear-explosive (CBRNe) events management is one of the most complex sets of analysis, procedures, rules, and courses of actions that an organization has to follow in order to properly react to such a threat and minimize its consequences. Generally, every CBRNe event (accident/threat) can be divided into a pre-event phase and three post-event response and recovery phases. The phase preceding the accident/threat is preparedness and it is of primary importance: in fact, the more first responders and advisors are trained and experienced, the most effective the response will be, allowing operators to save more lives. After the event, the short-term, intermediate and long-term phases will follow. The short-term phase includes the notification and first response sub-phases in which the event is first notified, and then the local emergency services are gathered on the scene. During the intermediate phase, a characterization (in which every detail of the operation is made available), a decontamination (in which victims and personnel are treated outside the immediate danger zone) and a clearance (in which the area must be fully decontaminated in order to be “cleared” and go back to its common operational functions) sub-phases follow in order. Last, in the long-term phase, restoration and re-occupancy sub-phases make sure the site involved in the CBRNe event is recovered to pre-event conditions. Management of all these phases requires the most rapid and accurate determination of the distribution of the toxic agent. When it comes to accidents involving radioactive substances - the so-called TIR (Toxic Industrial Radiological) - properly equipped commercial drones can help to detect the sources and the contamination in a very short time; therefore, they are considered essential tools in the hands of today first response units in order to achieve the correct situation awareness (collecting data that can be used as input for CBRN information management software) and have advisors that provide decision-makers with the best course of action [5,6,7,8,9]. In this way, using source terms obtained in the field, the predictive software (e.g., HotSpot Health Physics Code, CBRN-Analysis, etc) can elaborate more accurate and realistic responses.

In this work the benefits of modeling and simulations approaches versus real experimentation on the field are first introduced, then a detailed approach to computational fluid dynamics based on the Navier-Stokes equations is presented; eventually, the modus operandi needed to fulfill the expected results is also described [10]. The computational software used in the modeling and simulations phases will be introduced along with the design of a virtual drone; the description of the subsequent Computational Fluid Dynamics (CFD) simulations, the collection of the results and their analysis for the above-mentioned goal will also be shown. The aim of this work is to provide a methodology based on numerical simulation to analyze and improve the performance of a drone with a radiological sensor to monitor dangerous areas. The conclusions will address the results of the overall work and propose a pathway for future fulfillments in the Nuclear-Radiological response field.

## 2. Materials and Methods

In this section the authors describe the methodology to evaluate and improve the performances of sensors sensitivity on a drone, taking into account the fluid-dynamics interaction through the software COMSOL Multiphysics^®^ (COMSOL Inc., Stockholm, Sweden). The authors provide an explanation of the advantages of using numerical simulation versus experiments followed by the case study analyzed in this work. The last two parts of these sections are devoted, respectively, to explain the models and the designed geometry.

### 2.1. Numerical Simulations vs. Experiments

The design of a commercial drone that hosts CBRNe sensors for detection and identification purposes requires a deep analysis of the aerodynamic forces generated by the drone propellers since they could influence the detection process and compromise the final results. In order to be positively sure that a specific design of a drone with sensors will be able to detect the target substance/particles during the approach/hoover/descent phases, many tests need to be performed and direct observations must be recorded. Direct experimentation could lead to the requirement of carrying out several cycles per trial, wasting not always available time and resources. Direct numerical simulations, exploiting high speed powerful digital computers, are able to solve approximations of the equations describing the physical process. In Computational Fluid Dynamics (CFD) several algorithms are used; in particular, in finite volume algorithms, the entire fluid domain is divided into several little cells in which Navier-Stokes equations, once provided with initial and boundary conditions, are solved using an iterative methodology. However, fluid-dynamics simulations are not direct, and they use models that introduce a relatively large error (turbulence models). The errors introduced by the models chosen are usually partially compensated by validating the results of the simulations in accordance with some selected experimental tests.

### 2.2. The TIR Scenario and Drone Modeling

The present study addresses the problem of the optimization of the sensor location on the drone to avoid data collection losses caused by the aerodynamic forces that could alter the sensor detection. The main focus is environmental detection for a small radioactive source i.e., detection of radioactive medical waste non-properly disposed of. The presented numerical simulation analyzes if and how the turbulence caused by the propellers flow could influence the data value of the sensors in relation to their positions on-board the drone. On real application, the system could be designed to give the first warning about a little disperse radiation source in the environment.

Many pieces of follow-up work have been conducted to study the effects of radiation on the electronic components. Such as in [11] for gathering radiation information in the event of a nuclear disaster, the goals were to develop a prototype radiation detection and mapping unmanned aerial vehicle system to safely identify irradiated areas in the event of a nuclear emergency.

The problem of the electronics components exposed to the ionizing radiation and the evaluation of sensors with the focus on the laser sensors has been carried out in [12]. Evaluation of sensors for mobile robots based on irradiation experiment in order to confirm and increase their anti-radiation performance for the mobile robots i.e., deployed in Fukushima nuclear power plant [13]. Due to the effects that radiation may cause on electronic components of mobile robots [14,15] studies have been carried out on the effects and the mitigation methods against them, which revealed the importance of establishing a knowledge-base about the way of designing radiation-tolerant or radiation-hard electronics systems even for terrestrial applications.

Monitoring of high doses of ionizing radiation such as that of Fukushima, for example, is not included in our analysis. Therefore, no significant effects are expected on electronic components subject only to small doses of radiation such as those to which this application is addressed. Moreover, the direction taken is to use low cost and commercial components as drones available on the market and low-cost sensors with the aim of having a detection just for a first warning. This implies that the hardware is not aimed at critical applications such as detection within a nuclear power plant.

The scenario analyzed for the following CFD simulations is an accidental Toxic Industrial Radiological release into the atmosphere of particles belonging to three different isotopes: Cesium 137 (β-γ emitter − half-life = 30 years), Cobalt 60 (β-γ emitter − half-life = 5.27 years) and Americium 241 (α-γ emitter − half-life = 432.2 years) [16]. The choice of the first two isotopes falls on their availability in hospitals for radiotherapy, while ^241^Am can be found in all common smoke detectors.

In general, these isotopes are subject to become orphan sources if not correctly registered and controlled and that is exactly what the scenario suggests: a certain quantity of the three isotopes has been abandoned by fault and enters into the process of a factory for special material recycling.

**Figure 1:**
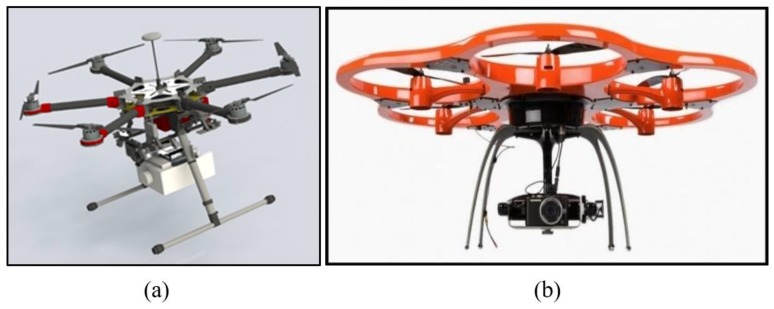
SR-FG6 hexacopter model (**a**) and a joint ducted propellers hexacopter (**b**).

In order to carry out the simulations, a 3D virtual drone needs to be designed, possibly after an existing prototype. The hexacopter has been chosen as the reference drone type because it represents the best performance compromise in terms of speed, stability, and aerodynamic control if compared with the quad-copter and the octa-copter. The starting prototype is the SR-SF6 hexacopter by Skyrobotics (Figure 1a).

In addition, it has been decided to include into the design ducted propeller rotors in order to allow for external stores connected to the outer edge of the ducts, as deemed necessary for the simulations. This concept would make the virtual drone look like another real prototype (in Figure 1b) with the exception that ducts are separated from one another to avoid the exposure of too many surfaces to aerodynamic forces.

**Figure 2:**
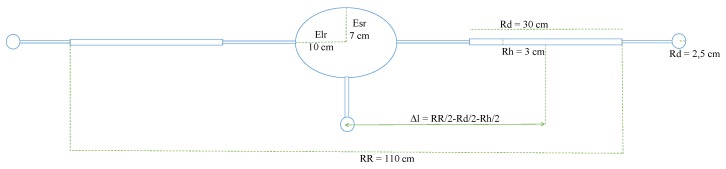
The side-sketch of the virtual drone prototype.

The virtual drone has been simplified for CFD simulations (Figure 2), without degrading the overall performances: an ellipsoidal central body connected to 6 engines with ducted propellers but no landing support.

### 2.3. Fluid Dynamics Model

The numerical simulations have been conducted using COMSOL Multiphysics software. A multiphase numerical model, based on a Euler-Euler approach, has been used to simulate the motion of the continuous (air) and dispersed (particles) phases.

The model used is a Euler-Euler approach. In the following equation, the continuum phase variables are indicated with the subscript “c” while the variables of the dispersed phase have the subscript “d”. The first differential equations are the conservation of mass of both phases:

(1) $$\frac{\partial \alpha_{c}\rho_{c}}{\partial t}+\nabla \left(\alpha_{c}\rho_{c}u_{c}\right)=0$$

(2) $$\frac{\partial \alpha_{d}\rho_{d}}{\partial t}+\nabla \left(\alpha_{d}\rho_{d}u_{d}\right)=0$$

where ρ_i_ and α_i_ are the density and the volume fraction of the i-th phase, while **u_i_** is the velocity vector of the phase. The total density of the mixture can be written as follows:

(3) $$\rho_{m}=\alpha_{c}\rho_{c}+\alpha_{d}\rho_{d}$$

The conservation of the momentum is taken into account through a set of two equations:

(4) $$\frac{\partial \left(\alpha_{c}\rho_{c}u_{dc}\right)}{\partial t}+\nabla \left(\alpha_{c}\rho_{c}u_{c}u_{c}\right)=\sum F_{c}$$

(5) $$\frac{\partial \left(\alpha_{\mathrm{cd}}\rho_{d}u_{d}\right)}{\partial t}+\nabla \left(\alpha_{\mathrm{cd}}\rho_{d}u_{d}u_{d}\right)=\sum F_{d}$$

where F_c_ and F_d_ are the forces applied to the continuous and dispersed phases. Gravity force apply to both the phases ($F_{g,i}=\alpha_{i}\rho_{i}g)$. The stress tensor, compounded by the pressure and deviatoric stress tensor, is computed for the continuous phase. The third force is the phase-interaction force, which takes into account how the continuous phase interacts with the dispersed phase and vice versa. Basically, this force considers the drag, the lift, and the virtual mass force. In the case of spherical particles, the lift is negligible, and the particle motion is dominated by the drag force. In the present simulation, the Schiller-Neumann drag model is used. The fluid is supposed to be isothermal.

The turbulence model used to simulate the effect of small scale effects is, instead, the k-ε, commonly used in these kinds of applications [17,18,19,20]. In the case of multiphase flow, the “mixture” quantities (taking into account both phases) are used to solve the turbulence equations.

(6) $$\rho \frac{\partial k}{\partial t}+{\rho u}_{m}\cdot \;\nabla k=\nabla \cdot ((\mu_{m}+\frac{\mu_{T}}{\sigma_{k}})\nabla k)+P_{k}-\;\rho \varepsilon$$

(7) $$\rho \frac{\partial \varepsilon }{\partial t}+{\rho u}_{m}\cdot \;\nabla \varepsilon =\nabla \cdot ((\mu_{m}+\frac{\mu_{T}}{\sigma_{\varepsilon }})\nabla \varepsilon )+C_{\varepsilon \;l}\frac{\varepsilon }{k}P_{k}-C_{\varepsilon \;2}\rho \frac{\;\varepsilon^{2}}{k}$$

where $u_{m}$ and $\mu_{m}$ are, respectively, the velocity and the viscosity of the mixture.

### 2.4. Choice and Setting of COMSOL Multiphysics Parameters

**Figure 3:**
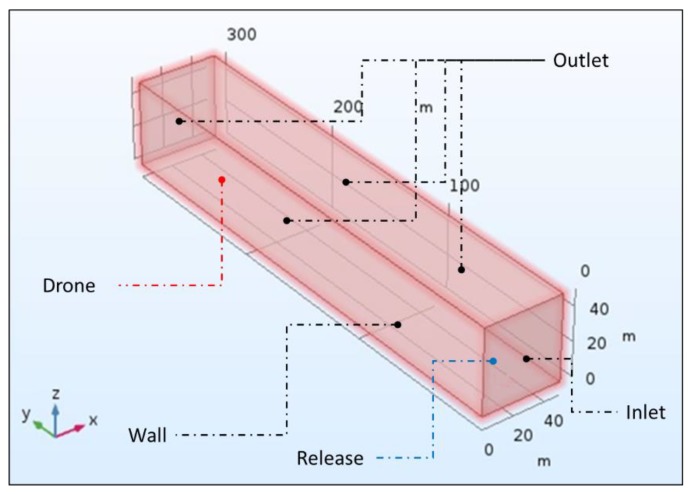
Schematics of the numerical simulation geometry.

In order to run a CFD simulation in COMSOL, the software needs to process the data of the main object of study—notably the drone—inside a specific domain with well-defined boundaries. The reference domain used is the following: a parallelepiped with a linear depth of 300 m (De), and a linear side (Si) equal to its height of 50 m (Figure 3).

Once the considerations on the virtual drone have been merged together with the sketch at Figure 1, it is possible to access the geometry builder to build each part of the drone by choosing its geometric shape and by providing the spatial information to the system (such as the reference semi-axis, the 3D coordinates inside the reference domain, the single component dimensions and the rotation angle if included).

**Figure 4:**
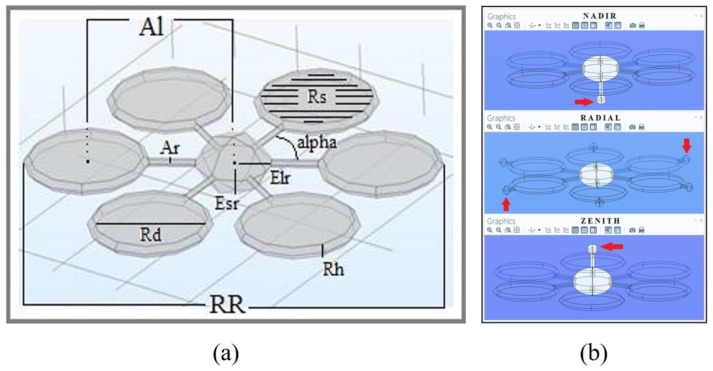
Schematics of the drone design with geometrical parameters (**a**) and positions of the sensors (**b**).

In the beginning, a three-element shaped body is created: an ellipsoid is selected for the drone central body, while two cylinders, the first one short and large and the second one long and thin, are used to model the rotor and its ducted propeller and the arm connecting the rotor to the central body respectively. Once these three geometries are built, a “union” function is used to glue the shapes into a single body. Finally, the drone is completed, except for the sensors, by maintaining the center of the ellipsoid as a fulcrum and applying a rotation on the XY plane by an alpha angle equal to 60 degrees for five times and gluing all the parts together (Figure 4).

The source of contaminant, in this case, a factory chimney releasing radioactive particles in the atmosphere, is represented in COMSOL by a solid sphere (see Figure 3 for the position) of 5 cm radius, dispersing, at the speed of 0.5 m/s, 1/100 mm diameter particles in concentrations of 3000 kg/$m^{3}.$ The dispersal phase volume fraction on the releasing sphere is 10^−9^.

Concerning the radiological sensor, it is necessary to run different simulations locating the detector in different spots of the drone. The virtual sensor is designed as a sphere with a radius of 2.5 cm in order to simplify the calculus (a slightly larger sensor would not affect the performance of the drone and the quality of the simulations). The simulations will run with three configurations of the virtual drone prototype, each one labeled based on the location of the detector. The Nadir drone hosts the sensor beneath the central body, the Radial drone hosts six sensors radially displayed on the outer edge of each propeller duct and the Zenith drone hosts it above the central body (Figure 4). Transforming the physical model into algebraical equations might induce significant errors in the results. To avoid this from happening, the surface is divided into sub-elements of geometrical simpler shapes (tetrahedrons), which are going to be studied one by one in order to increase the goodness of the approximation and to lower the magnitude of the final errors. The mesh settings determine the resolution of the finite element mesh used to discretize the model. The mesh used in a fluid flow simulation depends on the fluid flow model and on the accuracy required in the simulation. Generally, a fluid flow model requires a fine resolution in order to converge. The finest the resolution (number of subcells per unit volume), the highest the goodness of the approximation and the lowest the error on the results. In the following model, two different resolutions were used: a coarser one for the reference domain surfaces, and a finer one for the drone inside and the releasing sphere, whose dimension requires the sub-elements to be much smaller.

A mesh convergence analysis has to be performed in order to estimate the accuracy of the simulation: after running the simulation for the first time, the original mesh resolution must be doubled, and the simulation must be carried out once more. If the fundamental results change just within the expected tolerance, then the solution can be regarded as being mesh-converged. It is much easier, of course, to first identify the critical regions, and then to refine the mesh only before launching the simulation again.

Since the Euler-Euler Model will drive the simulations, the COMSOL setup phase requires to set the necessary input data. In the model, the dispersed phase consists of solid particles, while the reference pressure and temperature values are 1 atm and 293.15 K respectively.

In order to provide the continuous and dispersed phases with their rules of engagement, it is necessary to set the boundary conditions domain (Figure 3) for the virtual drone prototype and for the emitting sphere.

There is a total of 11 boundaries defined for the reference sphere (Figure 3):

⮚ The base surface of the reference domain is set as a wall: nothing can cross it.
⮚ The square surface at the origin of the reference domain is set as inlet (Inlet 1), and it allows the entrance of the continuous phase (the wind blowing at 3 m/s).
⮚ The upper surfaces of the drone rotors are set as outlet (Outlet 1) and represent the areas where the airflow exiting from the reference domain volume enters the drone rotors.
⮚ The lower surfaces of the drone rotors are set, individually, as Inlet 2 to 7, being the rotors exit flow and ingress, once more, in the reference domain volume.
⮚ The emitting sphere is set as Inlet 8, releasing the dispersed phase mixture into the reference domain volume.
⮚ The left, upper, right and opposite surfaces of the reference domain are set as Outlet 2 and allow the mixture to exit.

## 3. Results and Discussion

After completing the main set-up and before running the simulations, the last step was required: the virtual drone prototype and the releasing sphere had to be positioned inside the reference domain. The startup position setup was the following: the drone was at 250 m from the origin along the Y-axis, midway on the X-axis (25 m) and at a height of 10 m on the Z-axis, while the releasing sphere was at 15 m from the origin on the Y axis, same position of the drone on the X-axis and suspended at a height of 5 m on the Z-axis. The YZ plane crossing both the drone and the sphere had coordinates X = 25 m. This “position plan” holds the drone hovering 5 m higher than the releasing sphere at a relative distance of 235 m along the Y-axis.

Three simulations were carried out, one for each type of virtual drone prototype to test (namely the Nadir, the Radial and the Zenith). While the simulation is still running, a first check of the model variables can be performed, provided that the operator entered all the inputs correctly (garbage in garbage out—GIGO applies): in particular, the possible converging behavior of velocity, pressure, volume fraction of the dispersed phase, and turbulence variables can be observed by stabilizing a constant error value as the number of iterations increases. A difference up to 10^−3^ between the error values, if present, can be associated with the numerical model and the computational algorithms used, but it is not too relevant for the scope of the simulation.

**Figure 5:**
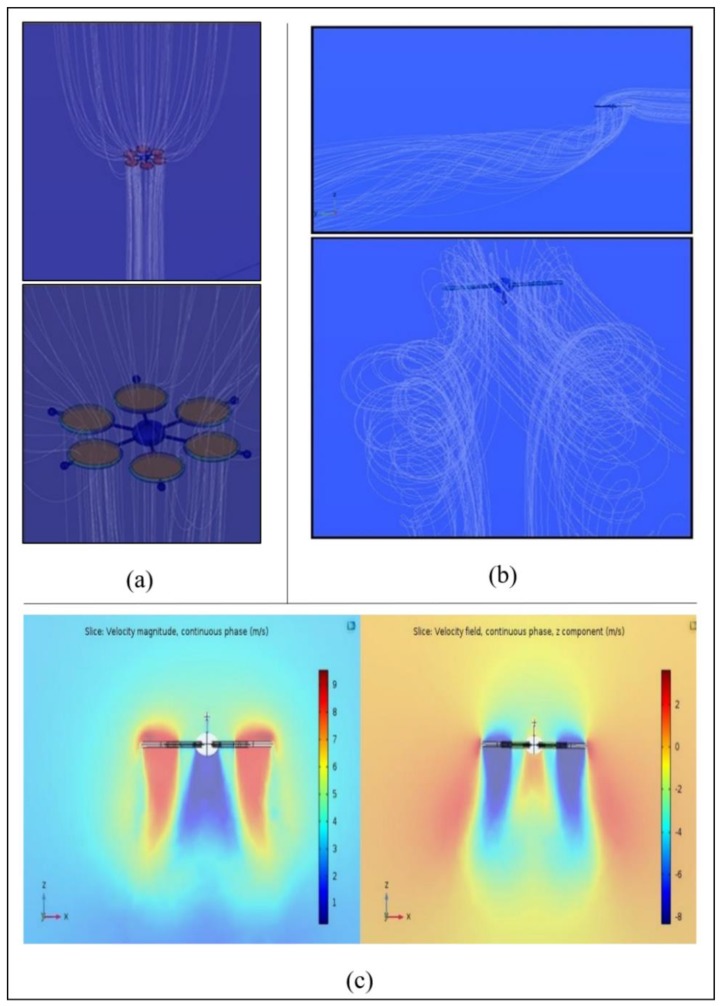
Air fluid flow lines expand before entering the drone domain, to account for the higher air amount requested by the engines and are concentrated downwards once re-entering into the reference domain (**a**); The same effect on the Nadir virtual drone prototype from two different points of view: as wind flow is absorbed by the upper side of the ducted propeller rotors is accelerated downwards. Re-interacting with the wind flow, the resulting vortexes are bent backward in a pattern very similar to that occurring at the aerodynamic flow leaving the wingtips of an aircraft during the flight (**b**); Velocity magnitude (left) and velocity along z-direction (right) near the drone (**c**).

The flow lines of the continuous phase are shown in Figure 5a. By examining the flow lines, it is also evident that the air-fluid is behaving in the correct way since air exits the reference domain into the upper side of the ducted propeller rotors to re-enter again the reference domain from the lower side. Using a small number of flow lines to show the fluid behavior (120 lines), COMSOL divides it by the number of propellers. It is important to note that the flow re-entering into the domain is accelerated and concentrated in a coherent downwash until the ground interference dissipates it radially, while the flow exiting the domain into the ducted propeller rotor upper surfaces tends to arrive not only from the direct vertical space but also from the outer boundaries of the ducts as the fluid starts its acceleration downwards and the mass flow request is higher. As the wind velocity is higher than the normal airflow in the reference domain portion above the drone, the airflow lines are shown to originate from the wind direction only. After entering the rotors, they are accelerated downwards and, still affected by the wind flow, they are twisted backward, as shown in Figure 5b.

The behavior of the drones seen from the plane crossing their longitudinal axis, which corresponds to the direction of movement, is very similar in terms of velocity of the continuous phase. The hexacopters move forwards with two parallel frontal rotors, followed by an external couple and by a rear one. The velocity below the rotors is slightly accelerated and two counter-rotating vortices are produced.

In order to determine the best location of the radiation detectors on the drone, it was primary to instruct COMSOL on how to calculate the particle concentration values at any given position in the volume fraction of the dispersed phase. For this purpose, a new variable was introduced both in the definitions and the settings fields of the model builder in COMSOL.

At this point, it was crucial to understand how the drone rotors downwash would affect the radioactive mixture. Moreover, it was necessary to assess the best drone configuration (in terms of sensor location) based on the values obtained from the simulated particle distribution and concentration.

In order to evaluate the parameters of interest, the authors decided to run four different sets of simulations for each prototype (Nadir, Radial, Zenith), based on the concepts of progressively decreasing the distance between the virtual drone prototype and the releasing sphere and pairing off the heights of the two elements in the reference domain. The simulation sets have been labeled with a sequence of three numbers indicating the height of the drone, the relative distance to the releasing sphere and the height of the sphere itself. In the following section, the results of the four sets of simulations are shown. Each set provides:

⮚ The section views of the reference domains, showing-on a logarithmic scale the radiological plume, with the relative positions of the drone and the releasing sphere;
⮚ A graph showing the concentration of the particles vertically below one of the three virtual drone prototypes (other graphs, showing similar values, are omitted), one at medium height, and a last one at ground level;
⮚ The graphs of particles concentration around each sensor;
⮚ The convergence graphs (third and fourth set only) for each virtual drone prototype.

### 3.1. Simulations Set-1 {10-235-5}

**Figure 6:**
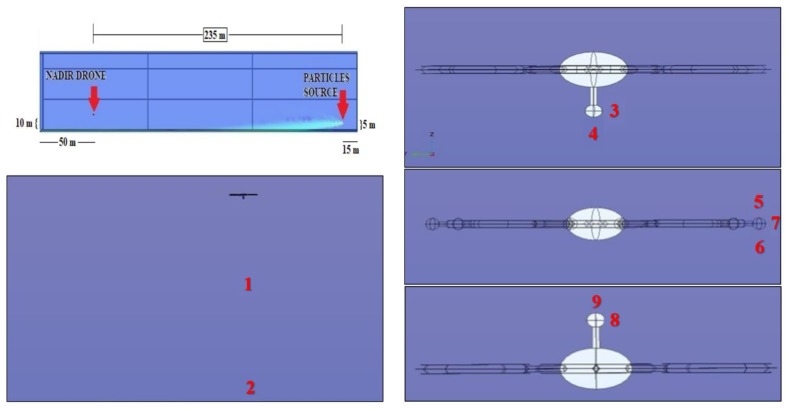
Schematics of the drone-sphere geometry, concentration field, and point analyzed in the numerical set 10-235-5.

The first numerical simulation has been conducted placing the drone at 235 m from the particle sources. The drone height is 10 m while the particle source height is 5 m. The plume of radioactive particles mostly falls down because of gravity (Figure 6) and the concentration near the drone is exactly zero. Thus, in this situation, all the sensor positions do not allow detection of the particles.

A quantitative analysis has been performed taking into account some points represented in Figure 6. The particle concentration in these points is shown in Table 1:

### 3.2. Simulations Set-2 {10-135-5}

**Figure 7:**
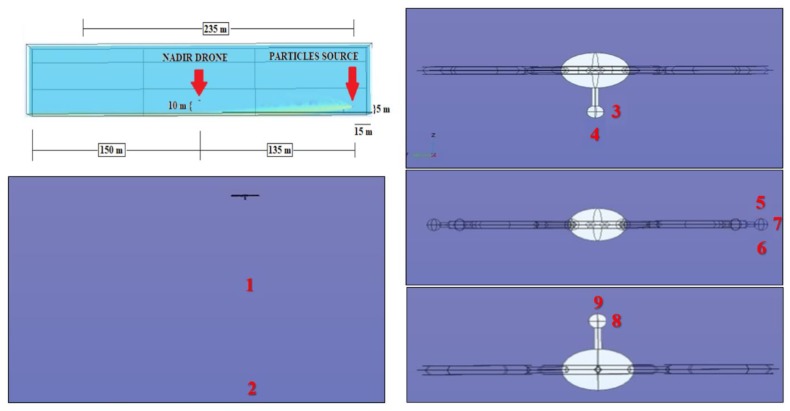
Schematics of the drone-sphere geometry, concentration field, and point analyzed in the numerical set 10-135-5.

The second numerical simulation has been conducted positioning the drone at 135 m from the sphere. Since the drone is closer to the emission source, the plume is still more concentrated, and few particles are still high. In this case, a particle concentration is larger than zero near the drone and in the three sensors positions, as indicated in Figure 7 and Table 2.

### 3.3. Simulations Set-3 {10-135-10}

**Figure 8:**
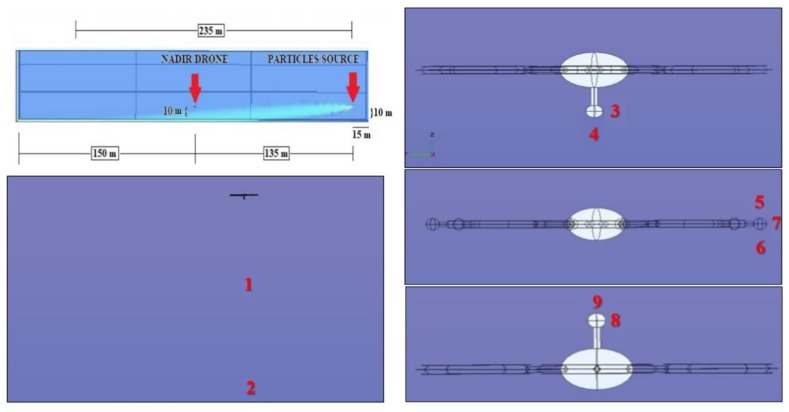
Schematics of the drone-sphere geometry, concentration field, and point analyzed in the numerical set 10-135-10.

The third numerical simulation has been conducted positioning the sphere at 10 m (Figure 8 and Table 3). The releasing sphere is now 5 m higher and at the same height as the drone. Because of the larger wind effect at this height, the plume tends to be more compact when flowing below the drone causing the sensors to be blind again. Also, the ground deposition of the dispersed phase has moved forwards from roughly 60 m to 220 m compared to that originated by the source at half-height, from roughly 30 m to 150 m.

### 3.4. Simulations Set-4 {10-100-10} 

**Figure 9:**
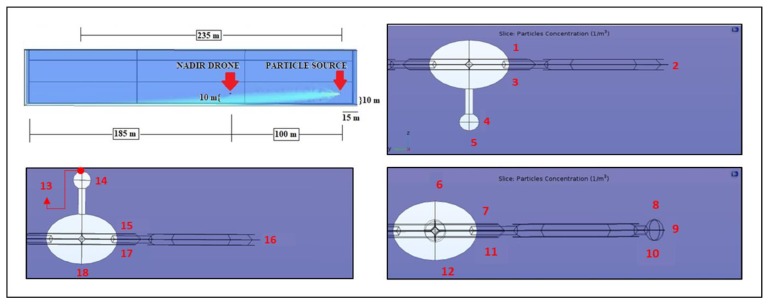
Schematics of the drone-sphere geometry, concentration field, and point analyzed in the numerical set 10-100-10.

The last numerical simulation has been conducted placing the drone at 10 m of height and far 100 m from the sphere source, which has a height of 10 m. In this case, high-particle concentrations have been detected in different positions of the drone (Figure 9 and Table 4). 

The radiological contamination from an elevated source, for example, the chimney of a factory following a TIR scenario, considers the radioactive particles as parts of metallic elements whose weight deflects the plume, bending it to the ground right outside the release point. The bending angle is influenced by the height of the release and the wind velocity component. Therefore, a drone equipped with detecting sensors for radiological detection is forced to fly not higher than the release source and close enough to detect, at least, the edge of the plume.

**Figure 10:**
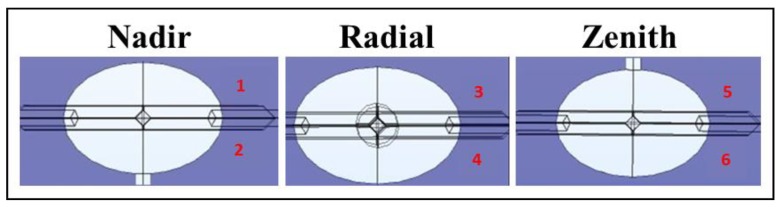
Reference points for the particle concentration comparison in the case of Nadir, Radial and Zenith solutions.

After four sets of CFD simulations, the best sensor performance was found using the configuration labeled as Nadir. This can be explained because the radioactive particles are able to penetrate the air barrier along the drone longitudinal axis (see the above picture for comprehension) and completely reach the drone sensor. Nevertheless, the best detecting results were obtained in front of the Nadir drone (Figure 10).

## 4. Conclusions and Prospects 

**Figure 11:**
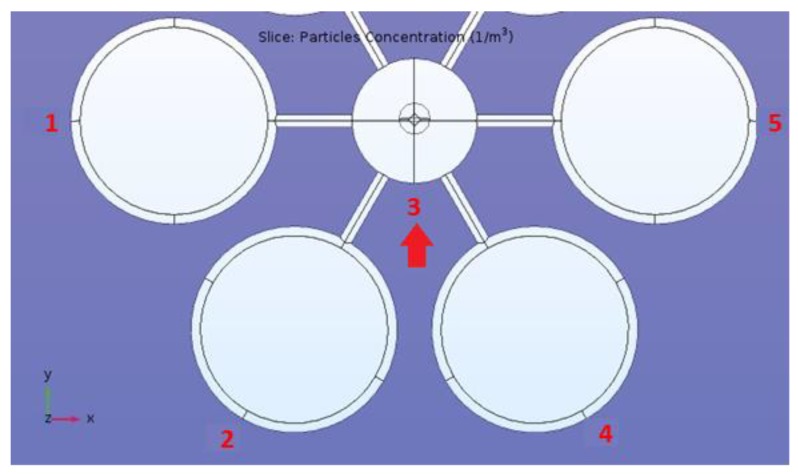
Reference points for the particle concentration as a function of wind direction.

The advantage of using numerical fluid-dynamics to explore and understand the behavior of different fluids, which usually require several models and different approaches, allows the researcher to perform troubleshooting procedures in order to find out if and where a model possibly failed or succeeded; moreover, sometimes numerical fluid-dynamics simulations may offer solutions beyond imagination. In this work, the results of the simulations helped to identify the best positions of a radiological/nuclear (RN) sensor on a drone in case of a TIR (Toxic Industrial Radiological) scenario. The calculated distribution of the concentration of the radioactive particles around the virtual drone prototypes brought unexpected results: the concentration of the particles was at the highest value always in the proximity of the drone central body, more specifically, in front of the ellipsoid along the movement direction. (Figure 10 and Figure 11, Table 5 and Table 6)

**Figure 12:**
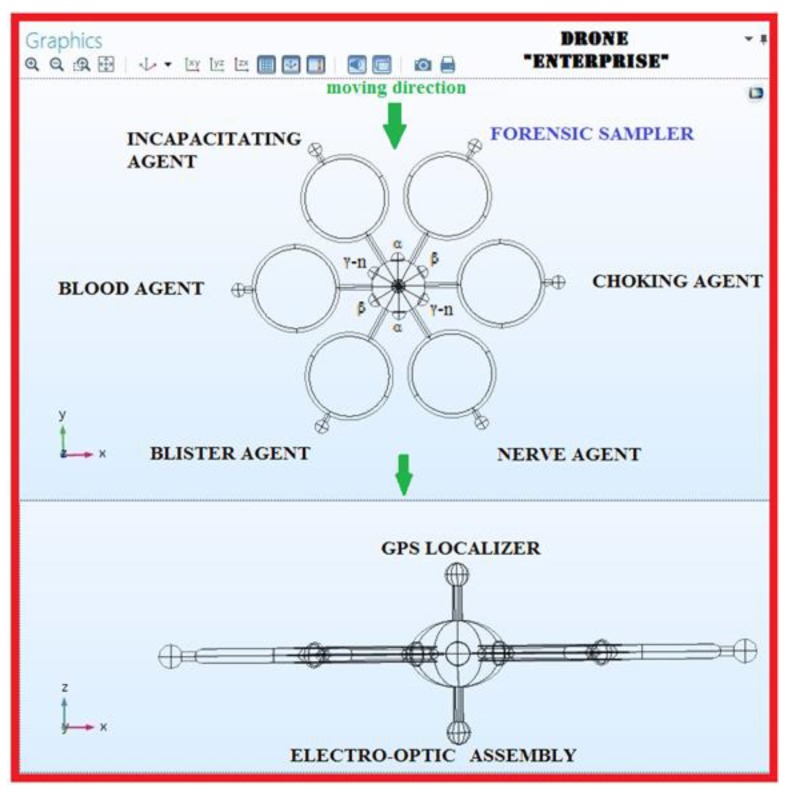
The “Enterprise” super detecting and identifying virtual drone design, showing all capabilities. Once Enterprise reaches the edge of a plume, it can be turned by 60° increments, while in hovering mode, to detect and identify many radioactive contamination, thanks to its sensors hosted at the maximum performance locations on the drone.

**Figure 13:**
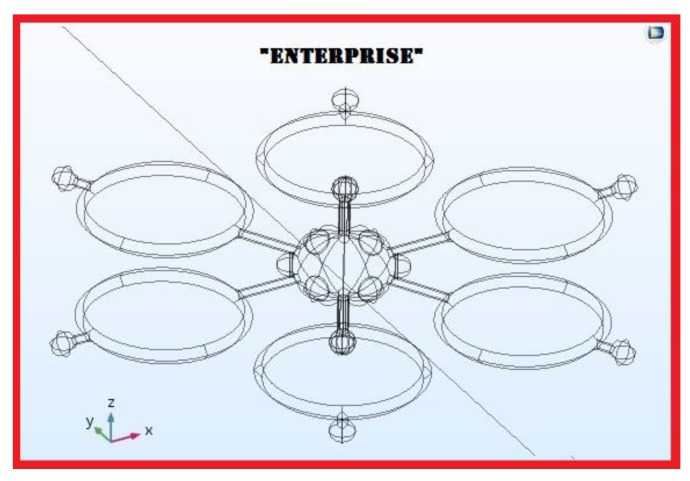
Enterprise, from a COMSOL 3D perspective.

The best virtual drone design, which may drive a successive realization step, for Radiological detection and identification is the Radial type with semi-submerged ellipsoid radial sensors; the same drone hosts also a GPS localizer at the zenith position and an electro-optical assembly for flight and recording at the nadir position. As introduced previously, the best candidates for the RN detection are γ-rays and β-particles; the type of radiation to be detected will drive the choice of the right category of ionizing radiation detectors to be mounted on the drone itself. In Figure 12 and Figure 13, the resulting virtual drone prototype designed in COMSOL model builder, nicknamed “Enterprise” is shown. Based on the results of a former research by this group, dealing with the same challenge but from a chemical detection perspective, the “Enterprise “ drone prototype is shown equipped with both chemical and radiological sensors at their best performing positions. 

Commercial drones will make a difference in CBRNe situational awareness in the near future. For this reason, having selected a Toxic Industrial Radiological (TIR) scenario involving the accidental release of ^137^Cs mixed with ^60^Co and ^241^Am, we have run four sets of simulations using three different virtual drone designs (Nadir, Radial and Zenith, named after the position of the respective sensors) inside COMSOL software; the simulations were carried out using a methodology based on Computational Fluid Dynamics (CFD). The goal of the simulations was to demonstrate how numerical analysis, based on CFD, may help to identify the best location where RN sensors should be located on a drone in order to enhance detection and identification capabilities. The results, based on the concentration of particles calculated in all the spots of the entire reference domain, showed that a radiological plume tends to bend towards the ground at high speed; moreover, the radiological plume has the ability to penetrate the rotors air barrier and reach the sensor underneath the drone. The Nadir drone configuration is the best for radiological detection among those studied and tested, but it should be observed that there is even a better location for a radiological sensor to perform at its maximum possible level, which is found in front of the drone central body. A new commercial hexacopter, equipped with low-cost radiological sensors, could represent a giant leap forward in CBRNe events management. In fact, it could provide mission commander, in a very short time and without exposing operators, with crucial information to get a full situational awareness, fostering decision-making process and optimizing prioritization of courses of action.

Since the simulations were performed using well-defined parameters, the results found are valid only in conditions and configurations similar to the ones used during the analysis; therefore, a group of simulations should be conducted in the future introducing different boundary conditions into the model in order to draw more general conclusions and guidelines.

Our simulations showed the effects of a possible interference, induced by the drone rotatory wings, on a radioactive particle dispersion. In order to validate experimentally the fluid-dynamics simulations results, several test campaigns will be designed and carried out. The first group of measurements will be planned and conducted in order to check the goodness of the fluid-dynamics simulation results. An inert powder, having the same physical characteristics (diameter of the single-particle, concentration, speed, and dispersed phase volume) of the radiological mixture implemented in the models, will be mobilized in an open space appropriate for the drone class used during the simulations. By monitoring the dispersion of the powder in the air, the actual effect of the turbulence induced by the drone rotatory wings will be investigated; the gathered data will confirm if the position of the sensor labeled as Nadir is indeed the best one among the simulated configurations.

Following the first campaign of measurements, a post-processing analysis of the acquired dispersion data will be conducted. Since the radioactive powder has been modeled as a mixture of ^137^Cs (β and 662 keV γ-rays’ emitter), ^60^Co (β and 1.17–1.33 MeV γ-rays’ emitter), ^241^Am (α and 60 keV γ-ray’s emitter) radioactive sources, both β-particles and γ-rays are appropriate candidates for the detection in air (α-particles have a very short mean free path in air, therefore their detection is not considered feasible). Each particle belonging to the dispersed dust cloud may be seen as a single radioactive source characterized by anisotropic emission; therefore, by selecting appropriate specific activity (Bq/g) values for the chosen emitters, the dose distribution field generated by a single particle in the air can be calculated. By processing the dose field of a single particle and the dispersed powder data recorded during the previous experiments, it will be possible to model the total dose distribution generated by the dust cloud. The dose field information will then be used both to select suitable radiation detectors for the application (based on their sensitivity and detection efficiency) and to design a plan of measurements to test the functionality of the detector.

This work demonstrated that this methodology, applicable to different scenarios, types of sensors and environmental conditions, allows the improvement of the sensor system layout avoiding part of the experimental activities saving time, effort and money.

## Figures and Tables

**Table 1:** Particle concentration in the reference points of simulation 10-235-5.

Point number	1	2	3	4	5	6	7	8	9
**Particle concentration [m^−3^]**	1.01 × 10^−4^	0.00258	0.00	0.00	0.00	0.00	0.00	0.00	0.00

**Table 2:** Particle concentration in the reference points of simulation 10-135-5.

Point Number	1	2	3	4	5	6	7	8	9
**Particle Concentration [m^−3^]**	0.00	0.0022	0.0017	4.26 × 10^−4^	3.23 × 10^−4^	3.31 × 10^−4^	4.98 × 10^−4^	6.49 × 10^−4^	2.76 × 10^−4^

**Table 3:** Particle concentration in the reference points of simulation 10-235-10.

Point Number	1	2	3	4	5	6	7	8	9
**Particle Concentration m^−3^]**	0.012	0.048	0.00	0.00	0.00	0.00	0.00	0.00	0.00

**Table 4:** Particle concentration in the reference points of simulation 10-100-10.

**Point Number**	**1**	**2**	**3**	**4**	**5**	**6**	**7**	**8**	**9**
**Particle Concentration [m^−3^]**	0.0459	0.0035	0.0381	0.0068	0.0015	1.37 × 10^−5^	0.0078	6.62 × 10^−4^	6.69 × 10^−4^
**Point Number**	**10**	**11**	**12**	**13**	**14**	**15**	**16**	**17**	**18**
**Particle Concentration [m^−3^]**	1.83E-3	0.0349	4.11 × 10^−4^	1.61 × 10^−4^	2.86 × 10^−5^	0.0018	0.0025	2.91 × 10^−3^	7.94 × 10^−4^

**Table 5:** Particle concentration for the Nadir, Radial and Zenith configuration comparison.

Point Number	1	2	3	4	5	6
**Particle Concentration [m^−3^]**	0.0459	0.0381	0.0077	0.0349	0.00181	0.00291

**Table 6:** Particle concentration for Figure 11 reference points.

Point Number	1	2	3	4	5	6
**Particle Concentration [m^−3^]**	0.0459	0.0381	0.0077	0.0349	0.00181	0.00291
